# Systems approaches to study root architecture dynamics

**DOI:** 10.3389/fpls.2013.00537

**Published:** 2013-12-26

**Authors:** Candela Cuesta, Krzysztof Wabnik, Eva Benková

**Affiliations:** ^1^Institute of Science and Technology AustriaKlosterneuburg, Austria; ^2^Department of Plant Systems Biology, Flanders Institute for Biotechnology (VIB) and Department of Plant Biotechnology and Genetics, Ghent UniversityTechnologiepark, Gent, Belgium; ^3^Mendel Centre for Plant Genomics and Proteomics, Masaryk UniversityBrno, Czech Republic

**Keywords:** root system, lateral root, genetic screening, transcript profiling, genomics studies, systems approach

## Abstract

The plant root system is essential for providing anchorage to the soil, supplying minerals and water, and synthesizing metabolites. It is a dynamic organ modulated by external cues such as environmental signals, water and nutrients availability, salinity and others. Lateral roots (LRs) are initiated from the primary root post-embryonically, after which they progress through discrete developmental stages which can be independently controlled, providing a high level of plasticity during root system formation. Within this review, main contributions are presented, from the classical forward genetic screens to the more recent high-throughput approaches, combined with computer model predictions, dissecting how LRs and thereby root system architecture is established and developed.

The root as an underground organ is of vital importance for plant life. It provides anchorage to the soil, supplies minerals and water, synthesizes metabolites, and interacts with symbiotic organisms (Den Herder et al., 2010). The root system architecture is shaped by the environmental signals and other external cues, which modulate root growth direction and kinetics (Luschnig et al., 1998; Lavenus et al., 2013), as well as its surface by affecting root hair growth (Lan et al., 2013) and frequency of branching (Sanz et al., 2011). In particular, formation of lateral roots (LRs) is one of the key determinants of the root architecture with an eminent impact on the efficiency of soil exploitation. For example, nitrate, phosphate, or sulfate availability modulate both primary root growth as well as LR formation and outgrowth (Linkohr et al., 2002; Hubberten et al., 2012), demonstrating close interconnection between nutrient availability and root architecture. LR organogenesis (Figure 1) is a well-defined process with discrete developmental steps including (i) priming, (ii) initiation, (iii) LR primordia (LRPs) organogenesis, (iv) LR emergence, and (v) activation of the LR apical meristem (Laskowski et al., 1995; Malamy and Benfey, 1997). These distinct developmental phases are under specific control mechanisms, providing a high level of plasticity during root system formation. To shape the root architecture in response to various external cues, plant hormones play an important role of rapid endogenous signal mediators (López-Bucio et al., 2003; Malamy, 2005). The core of this hormonal regulatory network comprises two antagonistically acting molecules: auxin and cytokinin. Auxin as a key stimulatory factor triggers and coordinates LR organogenesis, while cytokinin interferes with both initiation and LRP organogenesis (Benková et al., 2003; Laplaze et al., 2007; Fukaki and Tasaka, 2009; Bielach et al., 2012a).

**Figure 1:**
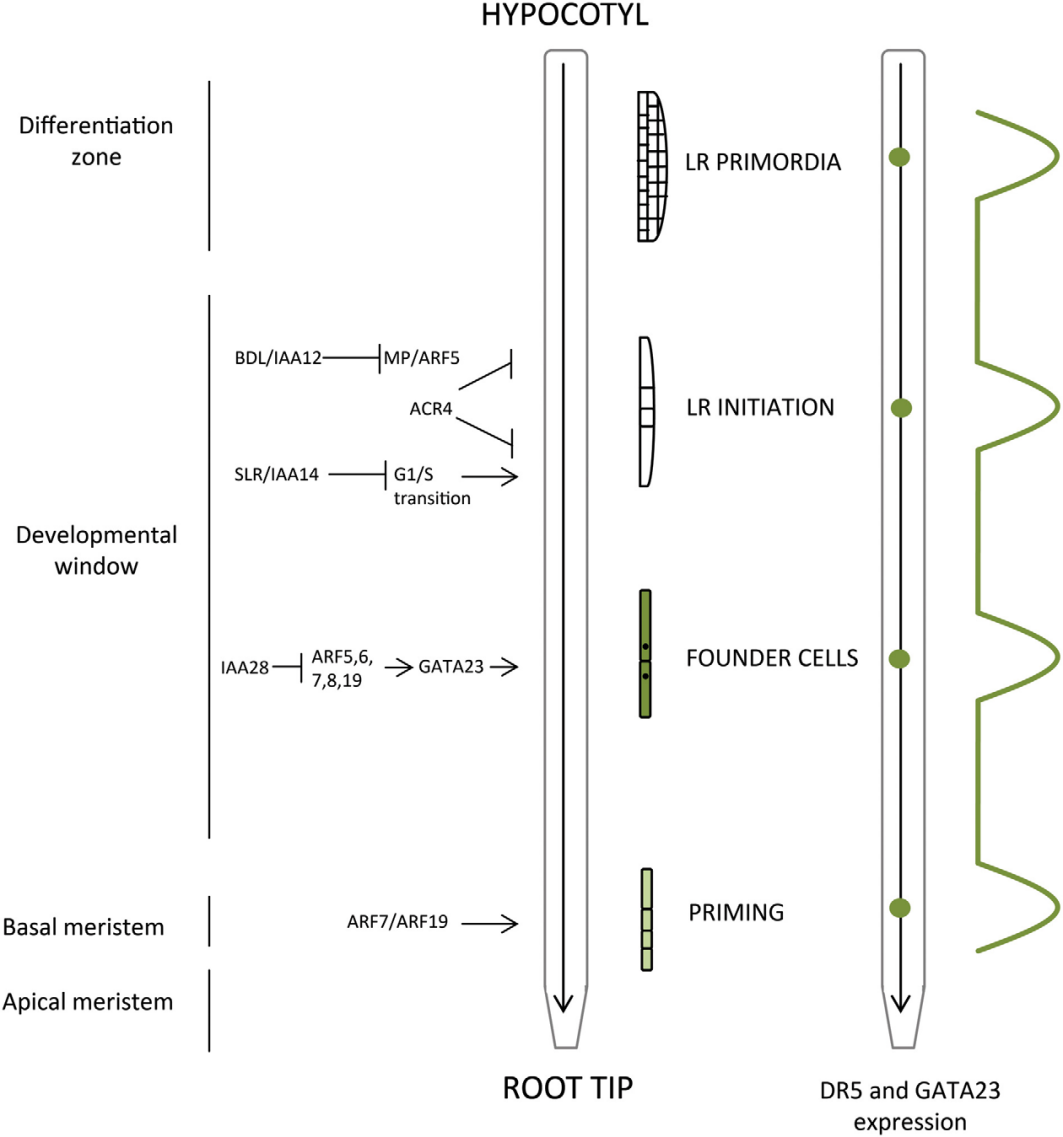
**Spatiotemporal control of lateral root organogenesis in primary root. Priming of LRP precedes LR initiation and occurs in the basal meristem**. Developmental window defines the occurrence of the FC establishment and most distal LRI, and LRPs develop and emerge through adjacent tissues in the differentiated part of the root. Correlation between the root zones (apical and basal meristem, developmental window, differentiation zone) with phases of LR development (priming, founder cells, LR initiation, LR primordia) along with the main molecular events are presented. The oscillatory mechanism that determines LR positioning as reported by DR5::LUC (auxin response marker) and GATA23 is depicted (green dots).

The exploration of the development of the root system has been confronted with various obstacles, such as the interplay between primary root growth and LR organogenesis, and technical challenges, such as limitations on isolating specific tissue layers of LRP which are hidden within tissues of the primary root. Therefore, studying root growth has required the development and implementation of specific tools and approaches. On other hand, some experimental advantages are obvious, e.g., there is a defined spatio-temporal frame or developmental window when cells are competent to initiate LRs (Dubrovsky et al., 2006, 2011), a capacity of ectopic induction and synchronization of LR initiation along the primary root by hormone application (Himanen et al., 2002), or easy-to-perform imaging of the LRPs.

Recently, root development research experienced great revival, especially because of an implementation of new generation imaging techniques and high-throughput approaches. The aim of this review is to acknowledge those techniques that have contributed to the deeper understanding of the development of the root system, as well as to present the most novel tools and their application potential. To present a broad picture of the formation and development of the root system we have discussed some of most prominent approaches for studying LR development that range from live-based methodologies to high-throughput technologies, combined with predictions from computer models of LR morphogenesis.

## Lateral root formation and development in light of real-time imaging

Implementation of the modern imaging techniques enabled to address longstanding questions on the LR organogenesis at qualitatively new level. In particular real-time imaging with high tissue resolution have provided a close spatio-temporal view on the early phases of lateral root initiation (LRI) and brought new insights into the dynamics of LR organogenesis.

In *Arabidopsis thaliana*, within the developmental window the position of the newly initiated LR is defined, i.e., the root zone where the cells exhibit the highest competence to initiate LRs (Dubrovsky et al., 2006). Early attempts to monitor LRI dynamics revealed that early events resulting in LRI correlate with regular fluctuations of an auxin sensitive reporter *DR5::*β *-glucuronidase* (*GUS*) in the protoxylem cells in the basal root meristem (De Smet et al., 2007). This recurrent enhancement of auxin activity, named priming, occurs with regular intervals of approximately 15 h, corresponding with the frequency of LRI during root growth. Later by the implementation of a real-time monitoring system using the *DR5::LUCIFERASE* reporter, more detailed studies of LRI dynamics could be performed (Moreno-Risueno et al., 2010). Luciferase imaging confirmed the oscillatory nature of the auxin response in the root, and correlated pulses of luciferase activity with prebranch sites that precede the establishment of static points where LRs are formed (Moreno-Risueno et al., 2010). Application of high-throughput transcriptome profiling synchronized with the oscillatory auxin response led to the discovery that besides auxin, a complex network of oscillating transcription factors contributes in setting up a prebranch site.

Another approach that combined the high end real-time imaging with computational modeling pointed out that the change in a shape of root cells as a consequence of gravitropic or mechanical bending redirects auxin flow toward pericycle cells, thereby resulting in an auxin accumulation in these cells that triggers LRI (Ditengou et al., 2008; Laskowski et al., 2008).

The next step in LR formation after priming and founder cell (FC) specification is the initiation of primordia organogenesis by series of anticlinal divisions. The identification of mutants in which despite FC establishment no LRI takes place (Celenza et al., 1995; DiDonato et al., 2004; Dubrovsky et al., 2008; De Rybel et al., 2010) indicates that the specification of FCs does not lead by default to LRI and that these two events might be uncoupled. Real-time imaging of LRI has shown that auxin continues to accumulate in the FCs, until it reaches a maximum just prior the actual initiation event (De Rybel et al., 2010). Later, an auxin reflux pathway, which is transiently established during the early phases of LRI, was found. This reflux via the PIN-formed (PIN3) auxin efflux carrier reinforces the auxin flow from the endodermal cells to the FCs, thereby enabling to reach the auxin threshold required to transit from founder to LRI phases (Marhavý et al., 2013).

LRP organogenesis continues by a series of cell divisions and differentiation coordinated by auxin. An auxin gradient with a maximum at the primordia tip is instructive for proper organ formation, and modulation of auxin distribution interferes with the progress of LR organogenesis (Benková et al., 2003). High resolution imaging of LRPs in real time turned out to be a powerful approach to bring further insights into the mechanisms controlling LRP organogenesis. 3D/4D image analysis (Lucas et al., 2013) revealed that early stage LRPs exhibit tangential divisions that create a ring of cells enclosing a population of rapidly dividing cells. The division patterns in the latter cell population during LRP morphogenesis are not stereotypical, although the shape of new LRPs is highly conserved. Interestingly, manipulating the properties of overlaying tissues disrupted LRP morphogenesis, indicating that the interaction with overlaying tissues might be more important for LRP morphogenesis than the precise division pattern.

Recently, monitoring LRP development when cytokinin is present revealed a rapid change in the PIN1 auxin efflux carriers expression caused by this hormone. Based on these observations cytokinin was proposed to interfere with LR organogenesis through regulation of the constitutive cycling of PIN1 by its alternative sorting to lytic vacuoles and subsequent degradation (Marhavý et al., 2011).

Similarly, understanding LR emergence and their interplay with surrounding tissues rapidly advanced with improved imaging techniques. In light of these observations it became clear that LR emergence is a tightly coordinated process during which auxin acts as local inductive signal to control cell separation in overlaying tissues (Swarup et al., 2008; Péret et al., 2012; Kumpf et al., 2013; Lucas et al., 2013). Moreover, applying real-time imaging improved our view on the acquisition of LR gravity sensing properties when emerging out of the parental root. The regulation of the gravitropic response of LRs, defining the gravitropic set-point angle, is crucial for the radial expansion of the root system (plagiotropism). Real-time analysis demonstrated that acquiring a gravity sensitive stage strongly correlates with the modulation of asymmetric auxin transport rates in LR columella, the differentiation of statoliths and the establishment of a connection to the primary root vasculature (Guyomarc'h et al., 2012; Rosquete et al., 2013).

## Genetic studies

A traditional strategy to identify the molecular components and mechanisms involved in a developmental process is forward genetics. Chemical agents (e.g., ethyl methanesulfonate) or radiation are used to induce mutations causing a certain phenotype, and using a mapping strategy the responsible gene can be identified (Lukowitz et al., 2000).

This approach has been successfully applied to reveal key components of LR formation (Table 1). Early screens based on the LR phenotype by Celenza et al. (1995) identified the *aberrant lateral root formation* (*alf*) mutants. Three different *alf* mutants were characterized: (i) *alf1-1*, with an increased number of LRs caused by IAA overproduction, turned out to be an allele of *SUPERROOT1* (*SUR1*) and *ROOTY1* (*RTY1*) [Boerjan et al. (1995) and King et al. (1995), respectively]; (ii) *alf3-1*, with arrested LRPs, that can be rescued by exogenous application of auxin; and (iii) *alf4-1*, unable to form LRs. Further work was done on the latter mutant by DiDonato et al. (2004), showing that ALF4 is required to maintain the developmental plasticity of pericycle cells and their meristem-like properties. Hence, *alf4-1* can perceive the LR induction signal but initiation cannot proceed because the xylem-adjacent pericycle cells cannot divide, since a mitotically active state is not maintained in the mutant. Another screen based on LR abundance has identified additional components specifically involved in the early phases of LRI, such as *Reduces Lateral root Formation* (*RLF*) (Ikeyama et al., 2010). The *RLF* gene codes for a cytosolic protein containing a cytochrome b5-like heme/steroid binding domain, and it seems to be involved in the activation of pericycle cell divisions at LRI sites downstream of auxin signaling.

**Table 1:** **Summary of the genes involved in LR initiation and development, including those summarized in Casimiro et al. (2003), De Smet et al. (2006) and Péret et al. (2009)**.

**Gene**	**Mutant**	**LR phenotype**	**Publication**
ABA1	*aba1*	Inhibition of LR	Vartanian, 1996; Signora et al., 2001
ABI3	*abi3-6*	Required for correct auxin response in LR	Brady et al., 2003
ABI4	*abi4*	Inhibition of LR	Signora et al., 2001
ABI5	*abi5*	Inhibition of LR	Signora et al., 2001
ACR4^*^	*acr4*	Increased LR	De Smet et al., 2008
AFB1	*afb1*	Decrease auxin response on LR	Dharmasiri et al., 2005
AFB2	*afb2*		
AFB3	*afb3*		
AGB1	*agb1-1, agb1-2*	Increased LR	Ullah et al., 2003
ALF1/SUR1/ RTY1^*^	*alf1-1/sur1/rty1*	Increased LR	Boerjan et al., 1995; Celenza et al., 1995; King et al., 1995
ALF3^*^	*alf3-1*	Arrested LR	Celenza et al., 1995
ALF4^*^	*alf4-1*	Lack of LR	Celenza et al., 1995; DiDonato et al., 2004
ARABIDILLO	*arabidillo1/2*	Reduced LR	Coates et al., 2006
ARF7/ARF19^*^	*arf7xarf19*	Lack of LR	Okushima et al., 2005
ARF8	*arf8-1*	Increased LR	Tian et al., 2004
	*Overexpression 35s::ARF8*	Reduced LR	
ARF 10/16/17	*Overexpression ARF10/16/17*	Reduced LR	Mallory et al., 2005; Wang et al., 2005
ARR3	*arr3*	Reduced LR	To et al., 2004
ARR4	*arr4*		
ARR5	*arr5*		
ARR6	*arr6*		
ARR8	*arr8*		
ARR9	*arr9*		
ATHB-2	*35s::ATHB-2*	Reduced LR	Steindler et al., 1999
	*reverse ATHB-2 sequence 35s::αATHB-2*	Increased LR	
ATHB-8	*Overexpression*	Reduced LR	Baima et al., 2001
AUX1^*^	*aux1* alleles	Reduced LR	Marchant et al., 2002
AXR1	*axr1-3, axr1-12*	Reduced LR	Lincoln et al., 1990
AXR2^*^	*axr2-1/iaa7*	Increased LR	Nagpal et al., 2000
AXR4	*axr4-1, axr4-2, axr4-1 axr1-3*	Reduced LR	Hobbie and Estelle, 1995
AXR6	*axr6-1, axr6-2*	Reduced LR	Hobbie et al., 2000
BRX2	*brx-2*	Increased LR (on cytokinin)	Li et al., 2009
CEG	*ceg*	Increased LR	Dong et al., 2006
CKX1	*Overexpression 35s::CKX1*	Increased LR	Werner et al., 2003
CKX3	*Overexpression 35s::CKX1*	Increased LR	Werner et al., 2003
DFL1	*dfl1-D, sense 35s::DFL1*	Reduced LR	Nakazawa et al., 2001
	*antisense 35s::DFL1as*	Increased LR	
E2Fa^*^	*E2Fa*	Reduced LR	Berckmans et al., 2011
ERA1	*era1-2*	Increased LR	Brady et al., 2003
ETA3	*eta3*	Reduced LR	Gray et al., 2003
GNOM	*gnom* alleles	Reduced LR	Geldner et al., 2004
GPA1	*gpa1-1, gpa1-2*	Reduced LR	Ullah et al., 2003
HAT2	*Overexpression*	Reduced LR elongation	Sawa et al., 2002
HOBBIT	*hbt* allele	LR meristem defect	Willemsen et al., 1998
HY5	*hy5-1, hy5-Ks50*	Increased LR	Oyama et al., 1997
IAA1	*axr5-1*	Reduced LR (on auxin)	Yang et al., 2004
IAA3^*^	*shy2-2*	Reduced LR	Tian and Reed, 1999
	*shy2-22, shy2-24*	Increased LR	
IAA14^*^	*slr-1*	Lack of LR	Fukaki et al., 2002
	*slr-1R1*	Poorly restored LR	
	*PICKLE/SSL2*	Partial restored LR	Fukaki et al., 2006
IAA18	*iaa18/crane*	Reduced LR	Uehara et al., 2008
IAA19	*msg2-1*	Reduced LR	Tatematsu et al., 2004
IAA28^*^	*iaa28-1*	Reduced LR	Rogg et al., 2001
IAR3	*ilr1 iar3 ill2*	Reduced LR	Rampey et al., 2004
ILL2			
ILR1			
ILR2	*ilr2-1*	Reduced LR	Magidin et al., 2003
KNAT6	*35s::RNAi*	Increased LR	Dean et al., 2004
KRP2	*35s::KRP2*	Reduced LR	Himanen et al., 2002
LAX3^*^	*lax3*	Reduced LR	Swarup et al., 2008
LIN1	*lin1*	No LR repression	Malamy and Ryan, 2001
MRP5^*^	*mrp5-1*	Increased LR	Gaedeke et al., 2001
NAC1	*Antisense 35s::NAC1*	Reduced LR	Xie et al., 2002
	*Overexpression 35s::NAC1*	Increased LR	
PAS1	*pas1*	Reduced LR	Faure et al., 1998; Vittorioso et al., 1998
PAS2	*pas2*	Increased LR	Faure et al., 1998; Bellec et al., 2003
PAS3	*pas3*	Reduced LR	Faure et al., 1998
PGP4	*pgp4*	Increased LR	Santelia et al., 2005
PIN1^*^	*Overexpression 35s::PIN1*	Delay LR development	Benková et al., 2003
PIN3^*^	*pin1 pin,3 pin3 pin7*	Reduced LR	
PIN4	*pin4 pin7, pin1 pin4 pin7, pin1 pin3 pin4*		
PIN7^*^	*pin1 pin3 pin7*		
PINOID	*Overexpression 35s::PID*	Reduced LR	Christensen et al., 2000; Benjamins et al., 2001
PLT1	*plt1 plt2*	Increased LR	Aida et al., 2004
PLT2			
PXA1	*pxa1*	Reduced LR	Zolman et al., 2001
RanBP1c	*Antisense AtRanBP1c*	Reduced LR	Kim et al., 2001
RAV1	*Overexpression*	Delay LR development	Hu et al., 2004
RCN1	*rcn1*	LR growth less NPA sensitive	Rashotte et al., 2001
RIB1	*rib1*	Increased LR	Poupart and Waddell, 2000
RLF^*^	*rlf-1*	Reduced LR	Ikeyama et al., 2010
RML1	*rml1*	Arrested LR	Cheng et al., 1995
RML2	*rml2*	Lack of LR	Cheng et al., 1995
ROP2	*CA-rop2*	Increased LR	Li et al., 2001
	*DN-rop2*	Reduced LR	
SBR	*sbr*	Reduced LR	Subramanian et al., 2002
SEU	*seu-3*	Reduced LR	Pfluger and Zambryski, 2004
SINAT5	*Overexpression 35s::SINAT5*	Reduced LR	Xie et al., 2002
	*Dominant negative 35s::SINAT5 (C49S)*	Increased LR	
SUR1	*sur1*	Increased LR	Seo et al., 1998
SUR2	*sur2/rnt1*	Increased LR	Delarue et al., 1998; Barlier et al., 2000; Bak et al., 2001
TIR1	*tir1-1*	Reduced LR	Ruegger et al., 1998; Dharmasiri et al., 2005
TIR3 (BIG)	*tir3-1, asa1/umb1*	Reduced LR	Ruegger et al., 1997; Gil et al., 2001; Kanyuka et al., 2003
WAK4	*DEX-induced WAK4 antisense*	Inhibition LR development	Lally et al., 2001
XBAT32	*xbat32-1*	Reduced LR	Nodzon et al., 2004
XPL1	*xipotl*	Increased LR	Cruz-Ramírez et al., 2004
YDK1	*ydk1-D, 35s::YDK1*	Reduced LR	Takase et al., 2004

* Genes discussed within this review.

Besides root oriented screens, investigation of other plant phenotypes (e.g., auxin defects, shoot appearance) has revealed remarkable components of LR formation and development. A screen for mutants defective in the shoot gravitropic response led to the identification of the *solitary root-1* (*slr-1*) mutant that completely lacks LRs (Fukaki et al., 2002). The mutation in *IAA14* belonging to the *Aux/IAA* auxin signaling repressor gene family stabilizes the IAA14 protein and as a consequence the auxin dependent initiation of LRs is disrupted. In efforts to reveal additional components of this SLR-mediated pathway, a suppressor screening was conducted on *slr-1*, identifying mutants such as *slr-1R1* (Fukaki et al., 2002) or *SSL2* (Fukaki et al., 2006). In the case of *slr-1R1*, an intragenic suppressor of *slr-1*, root hair formation is restored, but LR formation gets poorly recovered, indicating that LR and root hair formation require different mechanisms involving SLR/IAA14. The second mutant identified (*pickel/ssl2*) is an extragenic suppressor of *slr-1*. *PICKEL/SSL2* is a homolog of the animal chromatin-remodeling factor CHD3/Mi-2, implicating a role for chromatin remodeling mediated by PKL/SSL2 in the negative regulation of auxin-mediated LR formation in *Arabidopsis*.

Also screens targeting auxin signaling pathway resulted in the identification of mutants defective in LR organogenesis, highlighting the importance of auxin signaling in LR formation. That is the case for the auxin receptors TIR1 and related F box proteins AFB1, 2 and 3 (Dharmasiri et al., 2005). The loss of these genes resulted in a progressive decrease in auxin response during LR formation. Several Aux/IAA gain-of-function mutants, like *shy2*/*iaa3* (Tian and Reed, 1999) or *iaa28-1* (Rogg et al., 2001) exhibited dramatically reduced number of LRs, or like *axr2-1*/*iaa7* (Nagpal et al., 2000) showed an increased number of LRs. Similarly, the forward genetics approach has been employed to seek for new molecular components mediating the interaction between the auxin-cytokinin pathways during LR formation (Bielach et al., 2012b). Several *primordia on auxin and cytokinin* (*pac*) mutants in which the basal LRI process was not affected, but a cytokinin resistance phenotype appeared in the presence of auxin, might reveal new players balancing the auxin-cytokinin developmental output.

An alternative strategy is the reverse genetics approach, which is the analysis of mutants in genes selected based on prior knowledge about their role in specific pathways connected with LR formation. That is the case of the *Auxin Response Factor* (*ARF*) gene family, encoding transcriptional regulators that are core components of the auxin signaling pathway (Ulmasov et al., 1999). A PCR-based screening approach was conducted, identifying T-DNA insertions affecting the *ARF* genes (Okushima et al., 2005). By mutant phenotype characterization of several members of this family (specifically *ARF7* and *ARF19*), their role in LR formation was discovered. Similarly, by detailed mutant analyses the function of AUX1 and LAX3 auxin influx and PIN1, PIN3, PIN7, PGP1 and PGP19 efflux transporters in different phases of LR organogenesis has been recognized (Gaedeke et al., 2001; Marchant et al., 2002; Benková et al., 2003; Mravec et al., 2008; Swarup et al., 2008).

## Protein interaction studies

The lasting challenge in elucidating how LR formation is controlled is a complete dissection of the regulatory pathway components. DNA-protein or protein-protein interaction studies, such as yeast one-hybrid or yeast two-hybrid, are powerful approaches to uncover more new molecular players. By implementing this approach in the study of LR formation and development, a direct molecular link between auxin signaling, cell cycle machinery and LRI has been shown (Berckmans et al., 2011). The *E2Fa* transcription factor (regulator of cell cycle initiation) has been identified as a direct target of the *LATERAL ORGAN BOUNDARY DOMAIN18/ASYMMETRIC LEAVES2-LIKE20* (*LBD18/ASL20*) transcription factor downstream of auxin signaling and its role in triggering the first asymmetric division during LRI has been demonstrated (Berckmans et al., 2011). Additionally, by tandem affinity purification (protein-protein interaction) other proteins involved were identified, including LBD33. The data suggest that a LBD18/LBD33 dimer is necessary for *E2Fa* expression.

## Transcript profiling studies

Genome-wide transcript profiling is a high-throughput technology which enables the efficient evaluation of the complete transcript regulation in a certain process (Hennig et al., 2003). Besides particular genes, the identification and analysis of clusters of co-expressed genes might provide important insights on the physical or functional connection between gene products during the regulation of certain developmental process.

The true challenge in identifying regulatory genes involved in LR organogenesis by genome-wide profiling arises from the fact that LRI is restricted in time and space to a small number of pericycle cells hidden within surrounding primary root tissues. To circumvent this obstacle, a lateral root-inducible system (LRIS) was implemented to boost the frequency of LRI in a largely synchronized manner (Himanen et al., 2002). By combining LRIS with transcript profiling, Himanen et al. (2004) identified genes linked with early phases of LR initiation. Besides expected targets, such as components of the auxin signaling pathway and the cell cycle, clusters of regulatory genes co-regulated in course of the early phases of LRI were recognized. Later, using the LRIS set up, transcript profiles of the control and the lateral rootless *solitary root/iaa14* (*slr-1*) mutant were compared to extract genes linked with LRI (Vanneste et al., 2005). Within the genes identified, cell division-related genes were found (*APC8/CDC23*, *PCNA1*), directly linking auxin signaling and cell cycle activation during LRI both at the S-phase and the G2-to-M transition. Similarly, by extracting the auxin-regulated genes whose expression is strongly suppressed in the *arf7* and *arf19* mutants defective in LR organogenesis *LATERAL ROOT PRIMORDIUM1 (LRP1)*, *AUXIN-REGULATED GENE INVOLVED IN ORGAN SIZE* (*ARGOS*) or *LATERAL ORGAN BOUNDARIES DOMAIN* (*LBD*), family genes were uncovered for their role in LR organogenesis (Okushima et al., 2005).

Although the LRIS significantly increased the frequency of the LRIs, the limitation of tissue specificity was not overcome, since the material used included the whole root segment and contaminating tissues. Considering that a few cells within the pericycle layer are involved in a process such as LR formation, it is very likely that some important regulators could be missed. An elegant solution for this appeared to be an implementation of the Fluorescent Activated Cell Sorting (FACS) technique in combination with transcriptome profiling. To monitor the transcriptome of the xylem pole pericycle cells exclusively, the Gal4-GFP enhancer trap line J0121 with pericycle-specific expression was used (Laplaze et al., 2005). This improved strategy led to the identification of the membrane-localized receptor-like kinase *ARABIDOPSIS CRINKLY4* (*ACR4*), specifically transcribed in the small daughter cell after the first asymmetric pericycle cell division. The ACR4 is a key factor in promoting formative cell divisions in the pericycle, as well as in constraining the number of these divisions once organogenesis has been started (De Smet et al., 2008).

Later phases of LR development have also been targeted by transcript profiling studies (i.e., high-throughput quantitative RT-PCR). Namely, impact of the environmental signals, such as salt stress on primordia development has been examined (Duan et al., 2013). It was disclosed that the water stress-associated hormone abscisic acid (ABA) mediates suppression of LR emergence, acting primarily at the endodermis by tissue-specific ABA signaling pathways.

With the extensive increase of data generated by genome-wide profiling interesting targets might be easily overlooked. Hence, specialized algorithms and computational pipelines are developed to refine data mining and evaluation. A recently released spatio-temporal transcriptional map of the *Arabidopsis* root (the RootMap) (Brady et al., 2007) became an outstanding tool for evaluating expression patterns and gene correlations in root tissues.

A new tool named Visual Lateral Root Transcriptome Compendium (Visual RTLC) was developed by Parizot et al. (2010), in order to combine and compare the different datasets focused on LR organogenesis. These new appearing methods for data mining provide great opportunities to scale up the identification of novel regulators of LR organogenesis.

## Chemical genomics

Using the above mentioned approaches, crucial components of the LR regulation have been identified, and functional connections between key regulatory pathways (auxin, cytokinin, cell cycle-related), underlying root system architecture control, have been recognized.

Lately, the chemical biology opened new ways to study biological systems. The ability of chemical compounds to enhance, mimic, interfere or block a specific developmental process rises as a powerful tool to discover new regulatory components. The chemical approach is based on the ability of small synthetic molecules to modify the activity of proteins or pathways, resulting in the understanding of the protein function at a level that would be difficult to achieve through gene-based perturbation (Robert et al., 2009). Additionally, a tight temporal control can be accomplished, allowing for instance to overcome limitations related to mutational approaches (e.g., the long-term effect of disrupting the process can lead to lethality). Even more, its combination with other approaches, such as genomic studies, provides an additional power, i.e., the compound effect on a mutant that exhibits a certain phenotype.

The high-throughput screening of the chemical library must first be optimized in order to identify compounds that interfere with a specific developmental process. Aimed at LR organogenesis, an efficient screening method based on the LRIS was established (De Rybel et al., 2012; Audenaert et al., 2013). Using this platform, a naxillin, the non-auxin-like synthetic molecule that induces LR formation, was found as an activator of LRI, being more effective than known synthetic or natural auxins (De Rybel et al., 2012). A chemical approach combined with transcriptome profiling showed that 2581 vs. 401 genes are de-regulated by either auxin or naxilin, respectively, indicating a narrower mechanism of naxillin action when compared to natural auxin (De Rybel et al., 2012). Interestingly, genes involved in the early events of LR development such as *GATA23*, *LBD33*, and *LBD29*, were found to dominate in the naxillin induction profile. Forward genetics resulted in the identification of the *naxillin resistant 1* (*nar1*) mutation in the *IBR3* gene, linking naxillin activity with the regulation of the peroxisomal IBA-to-IAA conversion to promote the development of LRs.

## Computer modeling approaches

Computer models of plant development typically integrate experimentally identified interactions between genes and proteins (regulatory networks) to predict the dynamics of such regulatory networks in the developmental context (Prusinkiewicz and Runions, 2012). Numerous computer models of LR development have been developed to predict putative mechanisms underlying LR morphogenesis. These computer models often integrate experimental observations to identify a minimal mechanistic framework for LRI (Laskowski et al., 2008; Lucas et al., 2008). For example, mechanical deformation of cells was found to occur in the curved region of the primary root and to dramatically affect the size and shape of cells (Ditengou et al., 2008). Laskowski et al. (2008) demonstrated that a subtle change in the cell shape can be instructive for the auxin accumulation in pericycle cells and thus LRI. They also proposed that a feedback between auxin and expression of auxin influx carriers in pericycle cells further builds up this auxin maximum and thus promotes the LRI (Swarup et al., 2005; Laskowski et al., 2008; Péret et al., 2013). Hence, the combination of mechanical tension and auxin feedback on its transport can guide LR development in a self-organizing manner. On the other hand, a model developed by Lucas et al. (2008) suggests that root branching could be controlled by lateral inhibition—a different mechanism that depends on competition or distance between initiation sites and already emerged LR primordia.

Yet another approach attempted to approximate the complexity of sub-cellular regulatory networks that involve crosstalk between auxin and cytokinin that could influence both size and location of division and differentiation regions within the primary root as well as the putative periodicity of LR branching (Muraro et al., 2011, 2013). This type of modeling approach serves as a very useful tool to explore how dynamic response of auxin-cytokinin interaction network might change with respect to various mutant-like perturbations.

Finally computer modeling approaches have been applied to understand the physics and mechanics of LR development (Szymanowska-Pulka et al., 2012). Szymanowska-Pulka and colleagues reconstructed LRP morphogenesis based on anatomical observations and proposed a dynamic model of LRP growth that integrates acquisition of cell patterning that determines the final shape of the organ. Similar to that model the combination of live biological imaging, 3D/4D microscopic image reconstruction and dynamic computer model, have also revealed the relevance of coordinated patterning processes occurring in the proximity of the developing LRP that are central to the proper emergence of LRs (Lucas et al., 2013).

Taken together, a synergy of modeling and experimental efforts presented herein is likely to further generate new insights in LR patterning processes and ultimately broaden our understanding of the complex root system architectures.

## Novel tools and future perspectives

### Genetic studies: semi-automated phenotype analysis

Developing methods based on acquiring and analyzing developmental processes in real-time are continuously improving. Among others, the implementation of automated systems on root phenotype analyses combined with accurate images is a desirable feature. Fast and high-throughput phenotyping methods were developed to monitor the dynamic of root growth. For instance, the GiA Roots semi-automated software tool for high-throughput analysis of root system architecture (Galkovskyi et al., 2012), the semiautomated 3D *in vivo* imaging and digital phenotyping pipeline that enables high-throughput and accurate measurements of root system architecture through time (Topp et al., 2013), or the RootNav image analysis tool that allows the semiautomated quantification of complex root system architectures (Pound et al., 2013) were established.

Monitoring gene expression by live microscopy on a large number of specimens growing under controlled conditions to assess their spatio-temporal expression turns out to be another challenge. For this purpose, a microfluidic device (RootArray) where the roots are repeatedly imaged by confocal microscopy, coupled with an image analysis platform that includes automated real-time detection and tracking of samples, has been developed (Busch et al., 2012). This platform provides the ability to compare the reporter gene expression in *Arabidopsis* roots at tissue level in different developmental zones.

### Genetic studies: fast-forward genetics

A classical forward genetics approach implies the generation of a large mapping population, a high density of genetic markers for achieving a high resolution mapping, and the screening for recombinants in order to define the genetic interval where the mutation is placed (Lukowitz et al., 2000). Taking the advantage of Next Generation Sequencing, fast-forward genetics (SHOREmap pipeline) has been introduced, where the mapping is directly performed by sequencing (Schneeberger and Weigel, 2011). The SHOREmap pipeline covers from mapping to *de novo* marker identification during the sequencing process, and final annotation of candidate mutations (Schneeberger et al., 2009), hence profoundly increasing the efficiency of mutant identification.

### Transcript profiling studies: RNA sequencing

An improved high-throughput transcript profiling technology—RNA sequencing (RNA-seq), has appeared in the last years (Wang et al., 2009). This innovative technique allows the evaluation of the entire transcriptome. It can be used to determine the structure of genes, their splicing patterns and other post-transcriptional modifications, to detect rare and novel transcripts, and to quantify the changing expression levels of each transcript. When compared to microarrays, RNA-seq can detect all expressed genes without the generation of an array of probes, with reduced background noise and large dynamic range. This turned out to be particularly important in species such as tomato, where publicly available microarrays cover only one-third of the complete genome. The RNA-seq approach was used to analyze the transcriptome of tomato roots with the main focus on the spatial patterning and regulation of genes in the root by the hormones cytokinin and auxin. This transcriptome analysis of hormone regulation in tomato root revealed novel genes regulated by each of these hormones and can further be utilized as a reference to conduct future research on tomato roots (Gupta et al., 2013).

## Concluding remarks

Root system development is central for the plant to reach optimal growth. Hence, understanding the mechanisms that determine root architecture is of great agronomic importance, since they provide a basis for targeted engineering of plant architecture, e.g., for regulating root growth and branching to exploit less nutritious and arid soils. The availability of genome information has made it possible to study the gene expression on a genome scale, observing the behavior of many genes at a time, and obtaining a comprehensive, dynamic molecular picture. In the systems biology century, the only way to get insight in a developmental process is by combining synergistically the different available techniques, which include the most novel tools and advances. From this perspective, new available approaches are ready to be undertaken to obtain deeper insight in LR formation and development.

### Conflict of interest statement

The authors declare that the research was conducted in the absence of any commercial or financial relationships that could be construed as a potential conflict of interest.
